# Thermal Stability, Fire and Smoke Behaviour of Epoxy Composites Modified with Plant Waste Fillers

**DOI:** 10.3390/polym11081234

**Published:** 2019-07-25

**Authors:** Kamila Salasinska, Mateusz Barczewski, Monika Borucka, Rafał L. Górny, Paweł Kozikowski, Maciej Celiński, Agnieszka Gajek

**Affiliations:** 1 Department of Chemical, Biological and Aerosol Hazards, Central Institute for Labour Protection-National Research Institute, Czerniakowska 16, 00-701 Warsaw, Poland; 2 Institute of Materials Technology, Poznan University of Technology, Piotrowo 3, 61-138 Poznań, Poland

**Keywords:** polymer-matrix composites, plant waste fillers, thermal stability, fire and smoke behaviour, smoke toxicity

## Abstract

The influence of plant fillers on the flammability and smoke emission of natural composites was investigated. Epoxy composites with 15, 25, and 35 wt % of walnut and hazelnut shell, as well as sunflower husk, were prepared and examined. The ground organic components were characterized by grain size distribution, thermogravimetric analysis (TGA) and microstructure observations (SEM). The composite materials were subjected to dynamic mechanical analysis (DMA) and structural evaluation with scanning electron microscopy. Cone calorimeter tests and TGA determined the influence of plant waste filler addition on thermal stability and flammability. Moreover, the semi-volatile and volatile compounds that evolved during the thermal decomposition of selected samples were identified using a steady state tube furnace and a gas chromatograph with a mass spectrometer. The intensity of the degradation reduced as a function of increasing filler content, while the yield of residue corresponded to the amount of lignin that is contained in the tested plants. Moreover, the incorporation of agricultural waste materials resulted in the formation of a char layer, which inhibits the burning process. The yield of char depended on the amount and type of the filler. The composites containing ground hazelnut shell formed swollen char that was shaped in multicellular layers, similar to intumescent fire retardants.

## 1. Introduction

Interesting properties, high durability, aesthetics, as well as relatively low price all inspire a growing interest in plastics reinforced with lignocellulosic fillers, which are referred to as Natural Fibre Composites (NFC) or Wood Plastics Composites (WPC) in the case of wood fillers. Reports on polymers that were filled with plants, such as wood biomass, flax, hemp, jute, sisal, kenaf, and various types of cereals can be increasingly frequently found in the literature [1,2,3,4,5]. Construction and automotive industries are the main directions of application of these kinds of composites in the European Union. According to data, WPCs are mainly used in floorboards (67%), car equipment (23%), facades, terraces and fences (6%), technical applications (2%), furniture (1%) and consumer goods (1%). In the case of NFCs, almost 98% of the materials are applied in the automotive industry, mostly door and board components [6]. The properties of such fillers, in combination with environmental aspects, provide strong arguments for replacing synthetic fibres with natural ones. It is estimated that up to 30% of products that are made of polymers and glass fibres can be replaced with ones that are based on plant fillers [7]. 

Due to the fact that floor panels are flammable and they often trigger fires, it is required that the materials from which they are made are fire resistant [8]. At present, the flammability reduction of NFCs is accomplished by introducing as much as 60% of fire retardants [9]. Unfortunately, the proposed solution is not satisfactory to manufacturers, because of their non-ecological impact, negative influence on the properties, and higher costs.

Natural fibres that are exposed to flame or heat undergo thermal degradation and/or ignite, according to the conditions [10,11]. The mechanism of the thermal degradation process differs depending on the type of a given material and it is influenced by a number of factors, such as chemical composition, structure, degree of polymerization, and fibre orientation. The lignin decomposition process starts at the temperature range of 160–400 °C; weak bonds are broken at lower temperatures, while at higher ones ether and carbon bonds crack. The cyclisation process favours the formation of a char layer, which in the case of lignin is far greater than that of cellulose or hemicellulose. Low lignin content in fibre increases the decomposition temperature, and it reduces fibre resistance to oxidizing agents, which is related to the amount of aromatic groups. The degradation of hemicellulose, low mass polysaccharides, starts at 180 °C, causing a release of inflammable gasses and tar. An exothermic reaction takes place in the temperature range of 200–260 °C, which is associated with an increased emission of gaseous products of decomposition, tar generation, and local ignitions of low mass carbohydrates. Cellulose decomposes at above 260 °C generating a load of gaseous products, char (pyrophoric carbon), and tar. The presence of water may catalyse the decomposition process of cellulose. The decomposition process of natural fibres consists of a series of reactions:

- dehydration (evaporation of water);
- depolymerization and decarboxylation combined with dehydration of cellulose chains (formation of dehydrocellulose);
- decomposition of dehydrocellulose related to the formation of char;
- formation of levoglucosan;
- decomposition of levoglucosan to low mass, volatile gasses [12,13].

The process takes place over three most important phases: initial (flameless), main (flaming phase), and final (flameless) [14,15]. The initial phase includes a dehydration process with release of water. Up to 200 °C inner fibre bonds weaken and the material starts to release flammable gasses. The main phase includes ignition of the decomposition products and fire spread, which is accompanied by an increase in the heat release rate due to the progressive decomposition process and fibre mass loss. The phase occurs in the temperature range of 260–450 °C depending on the material. The final phase is associated with a slow extinguishing process and a formation of solid residue. 

It has been found that the course of burning of NFCs may be influenced, among others, by moisture content or morphology of the composites, as well as by the chemical composition and filler content [8]. Unfortunately, there are still only a few publications on the flammability of polymer composites modified by plant fillers other than wood, and they rarely discuss the problem of smoke emission. 

In our study, composites that are based on epoxy resins with agricultural waste material in the form of ground walnut (WS) and hazelnut shell (HS), as well as sunflower husk (SH) were prepared. Natural composites containing between 15–35 wt % of fillers were subjected to a series of DMA and TGA, as well as cone calorimeter measurements. The microstructure analysis complemented the influence of ground shell and husk on the properties of the composites. Additionally, a steady state tube furnace was used to determine the nature and quantity of the substances, which can evolve during the combustion and thermal degradation processes of natural composites. The main objective of the study was to manufacture thermoset composites that were modified with a plant waste filler of reduced flammability and smoke emission.

## 2. Materials and Methods 

Epoxy resin, based on bisphenol A (Epidian 624, epoxy number 0.48–0.51 mol/100g, viscosity 600–800 mPa∙s), and isophorone diamine curing agent (IDA, amine number 250–350 mg KOH/g, viscosity 150–300 mPa∙s), both produced by CIECH Sarzyna S.A (Nowa Sarzyna, Poland), were used as the polymer matrix. Composition ingredients were proportioned by weight: 50g of curing agent for 100g of epoxy resin, while following the producer’s suggestion. Walnut shell (Juglans Regia species, WS) and hazelnut shell (Corylus avellana species, HS) that were obtained from AGRO Jarosław Seroczyński (Nadbrzez, Poland), as well as sunflower husk (Helianthus annuus L. species, SH) from FODER BIO-TECH (Dębowa Góra, Poland), were used as agricultural waste fillers [16,17]. 

The grinding of the fillers was conducted on a laboratory sieve mill type MUKF-10 from Młynpol P.P.H. (Cerekiew, Poland). The mixing of the components was performed using a high-speed mechanical stirrer with a water jacket (Central Institute for Labour Protection-National Research Institute, Warsaw, Poland); three rotational speeds equal to 7000, 10,000, and 17,000 rpm, for 3, 1.5, and 0.5 min, respectively, were applied. Next, the components were degassed in a vacuum dryer for at least 1h and a curing agent was introduced. The mixtures were stirred again for two minutes at a speed of 7000 rpm and degassed for about 10 min. The samples were cured in forms at room temperature for 2 ± 1 days and then at 80 °C for 3 h. Three composite series with the fillers content equalling to 15, 25, and 35 wt %, as well as a reference material of unmodified polymer matrix were manufactured [16,17].

The particle size distribution of ground shell and husk was defined by means of a vibratory sieve shaker AS 200 from Retsch GmbH (Haan, Germany), which was equipped with sieves with the following mesh size: 0.02, 0.032, 0.063, 0.071, 0.125, 0.25, 0.5, and 0.8 mm. The measurements were carried out on a sample with the mass of 50 g over 1h. 

The surface structures of plant fillers and composites were based on the analysis of images from the scanning electron microscopy (SEM) SU-8010 from Hitachi High-Technologies Corp (Tokyo, Japan). Prior to the tests, all of the specimens of husk and shell were placed on the carbon tape and covered with a layer of gold while using sputter coater SC7620 from Quorum Technologies Ltd (Lewes, UK). The electron accelerating voltage of 5 or 10 kV was applied and the magnifications of 100× and 2000× were used in the case of filler, 400× for composites, and 100× for char residue. Thermo Scientific NORAN System 7 performed the analysis of the elemental composition of the char in FESEM, which was equipped with an electrically cooled Silicon Drift Detector EDS from Thermo Fisher Scientific (Waltham, MA, USA).

The dynamic mechanical-thermal analysis (DMTA) experiments were performed while using Anton Paar MCR 301 rheometer (Graz, Austria), equipped with a torsion DMA measuring system. The investigations were conducted with a constant frequency of 1 Hz and a strain of 0.01%. All of the samples were evaluated in the temperature range from 30 to 140 °C with a temperature ramp of 2 °C/min.

The thermal stability of the filler, the unmodified epoxy resin, and the epoxy composites were determined by TGA that was carried out in the temperature range of 30–900 °C at the heating rate of 10 °C/min under air or nitrogen atmosphere, using a TG 209 F1 Libra apparatus from Erich NETZSCH GmbH & Co. Holding KG. (Selb, Germany). Holding KG. 5 ± 0.1 mg samples were placed on ceramic pans. 

The burning behaviour of the composites was examined by cone calorimeter tests from Fire Testing Technology Ltd. (East Grinstead, UK), being conducted in accordance with the ISO 5660 standard. The specimens with dimensions of 100 mm × 100 mm × 10 mm and the weight of approx. 60 g were horizontally irradiated at a heat flux of 35 kW/m^2^. An optical system with silicon photodiode and a helium-neon laser enabled continuous measurement of the optical density of the smoke. Spark ignition was used to ignite the pyrolysis products and the values are the average obtained for three samples from each material.

**Figure 1:**
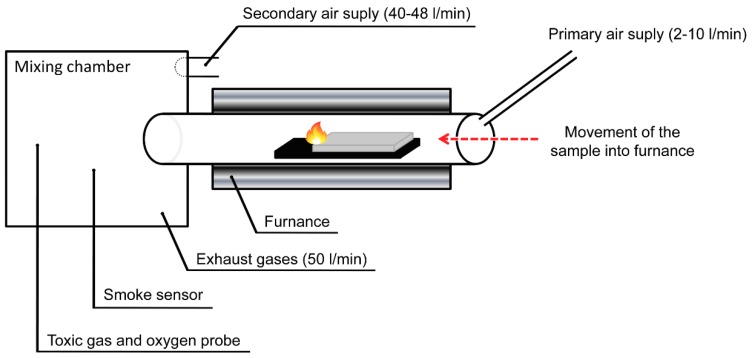
The scheme of steady state tube furnace (Purser furnace), adapted from [18].

The nature and quantity of the thermal degradation products of selected composites based on epoxy resins with agricultural waste materials were determined using a steady state tube furnace (Purser furnace) and a gas chromatography with a mass selective detector (Agilent Technologies 7890 A GC MSD 5975, Santa Clara, CA, US), in accordance with the ISO TS 19700 standard (Figure 1). The 5 g samples of materials were combusted at 650 °C. The fire effluents were taken from the mixing chamber of the tube furnace using the solid phase microextraction technique and carboxen/polydimethylsiloxane fibre coating, since it is recommended for the extraction of both non-polar and polar chemicals. The chromatographic separation was achieved with an HP-5 MS fused silica capillary column. Chromatographic peaks were identified through comparing the mass ions of each peak with NIST MS Library. As the chromatographic peak area of a specific compound is linearly correlated with its quantity, its concentration can be reflected by the peak area ratio. The summed identified peak areas were normalized to 100% and the relative abundance of a specific compound can be reflected by its peak area ratio.

## 3. Results

### 3.1. Natural Fillers Analysis

**Figure 2:**
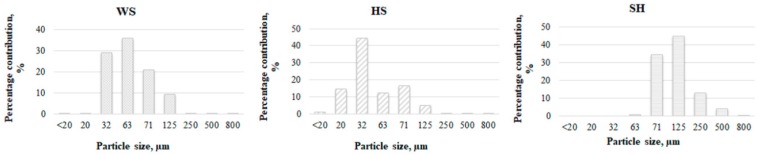
Particle size distribution by weight of ground ground walnut (WS), hazelnut shell (HS), and sunflower husk (SH).

Figure 2 presents the grain size distribution of ground shell and husk in relation to the mass of material that remained on individual sieves. It was found that 86% of the particles of WS and 73% of the particles of HS ranged between 32 and 71μm in size. The highest share of relatively large grain, for which as many as 62% of particles were above 71μm, was determined for ground SH. The most uniform grain size distribution, with more than 99% of the particles between 32–250 μm, was recorded for WS [16]. Nevertheless, it can be assumed that a fairly homogeneous size distribution characterizes all of the investigated fillers.

**Figure 3:**
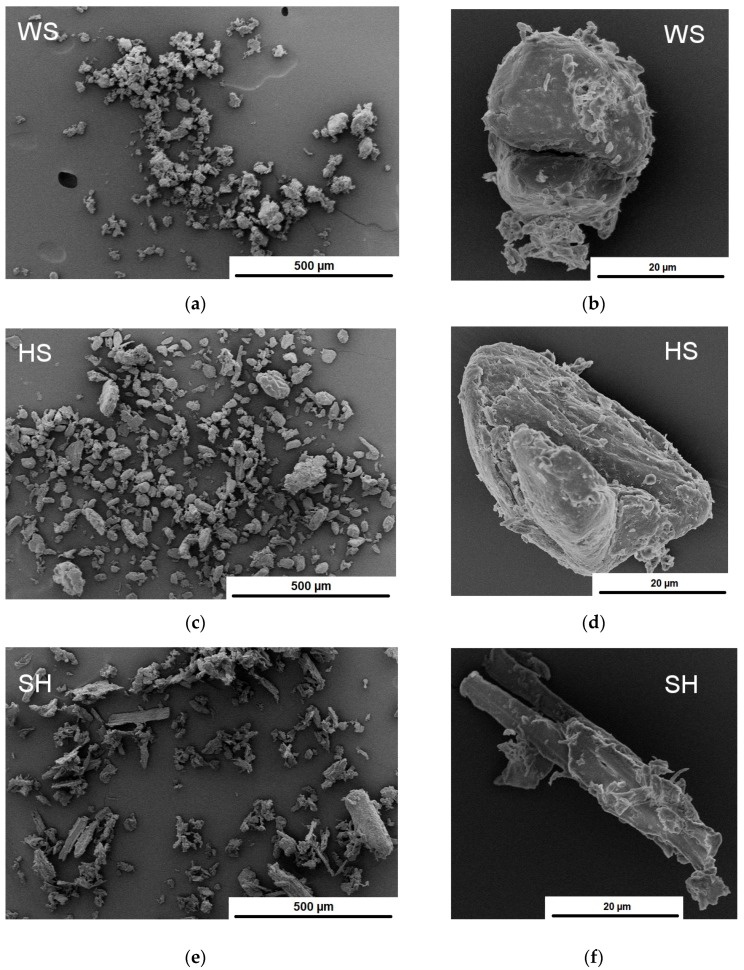
SEM images of ground WS (**a**,**b**), HS (**c**,**d**), and SH (**e**,**f**).

The morphology of the particles of the analysed natural waste plant fillers was assessed based on SEM images. The analysis of SEM images (Figure 3) confirmed that the particles that were obtained as a result of grinding WS, HS, and SH differed in size (Figure 3a,e) and geometry (Figure 3c). The greatest differences in the appearance of particles were observed between shell and husk, due to the different construction of such complex structures and their behaviour during the grinding process. The particles of ground shell resembled a sphere (Figure 3b), and moreover their surface was rough to varying degrees. In contrast, the particles of ground SH looked like short fibres, due to their longitudinal shape. Surface roughness impedes its proper wetting by the polymer matrix in the course of processing, thereby contributing to the formation of voids at the interface. The problem mostly refers to polymers with high viscosity. On the other hand, the well-developed surface of the particles increases the contact surface of the components and it enables their mechanical linkage [19,20,21].

### 3.2. Structure of Composite Materials

**Figure 4:**
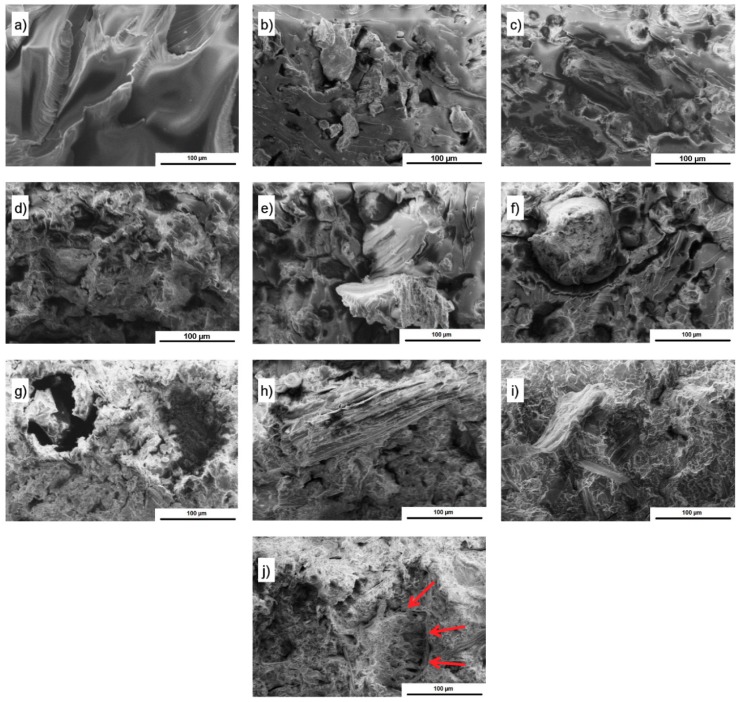
SEM images of unmodified epoxy resin (**a**) and composites with 10, 25, and 35 wt % of ground: WS (**b**,**c**,**d**), HS (**e**,**f**,**g**), and SH (**h**,**i**,**j**).

Figure 4 shows images of the unmodified epoxy resin as well as WS, HS, and SH composite fractures at 400× magnification. The analysis of SEM images allowed for observing the occurrence of the particles of fillers in the entire volume of samples, as well as their uneven arrangement and variation in size, in accordance with the SEM images of the grains before they were introduced into the resin (Figure 3). Uneven distribution of plant fillers in the polymer is characteristic for composites that were filled with particles or short fibres [22]. A proper wettability of the surface of the fillers resulting from the use of a polymer with a relatively low molecular weight increases the adhesion between the composite components [16]. It is not only that the surface of the filler is covered by the polymer, but it appears that the epoxy resin also penetrates into the porous structure of the particles. This phenomenon was visible, especially in the case of composites with SH (Figure 4j). Nonetheless, a considerable number of voids distributed in the epoxy matrix as well as in the region of components contact was found. The highest porosity was observed in the case of a series of composites that were modified with SH, regardless of the amount of the filler. Voids of significant size that occurred in the whole sample, whose share grew with the increase in the filler amount, might be the result of an impeded degassing of the highly viscous mixtures [16,17]. The particles of ground shell and husk differ in the aspect ratio and surface area [17], which affects the viscosity of composition. However, the voids located in the interphase region predominated for WS and HS composites, reflecting the limited adhesion between matrix and plant filler in the form of ground shell. This is also confirmed by the intact surface of the particles of these fillers, which are visible on fracture images. 

### 3.3. Dynamic Mechanical Analysis

**Figure 5:**
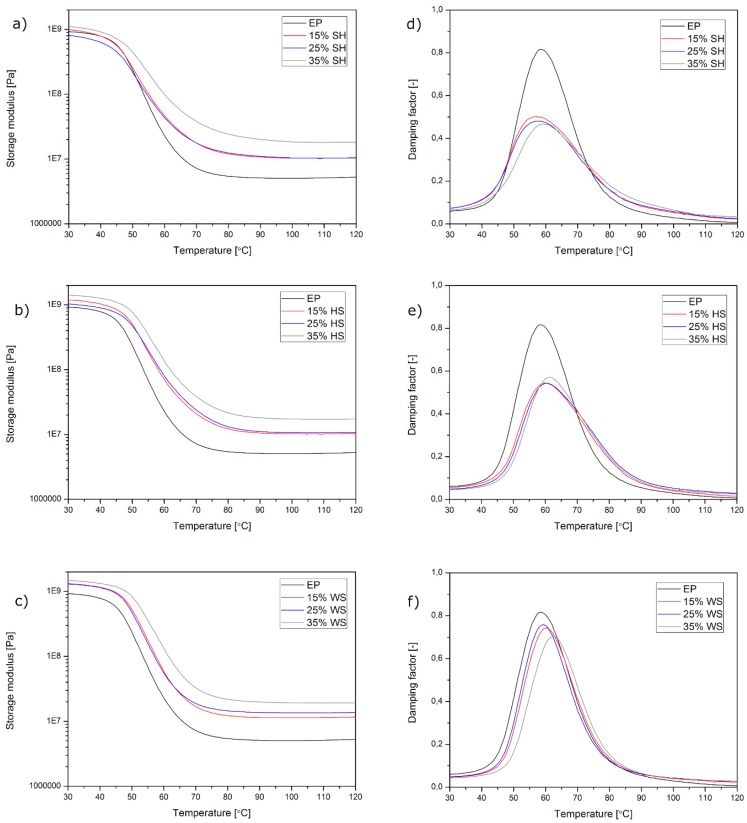
Storage modulus (G’) (**a**–**c**) and damping factor (tanδ) (**d**–**f**) as a function of temperature of pure cast epoxy sample and epoxy-based composites.

**Figure 6:**
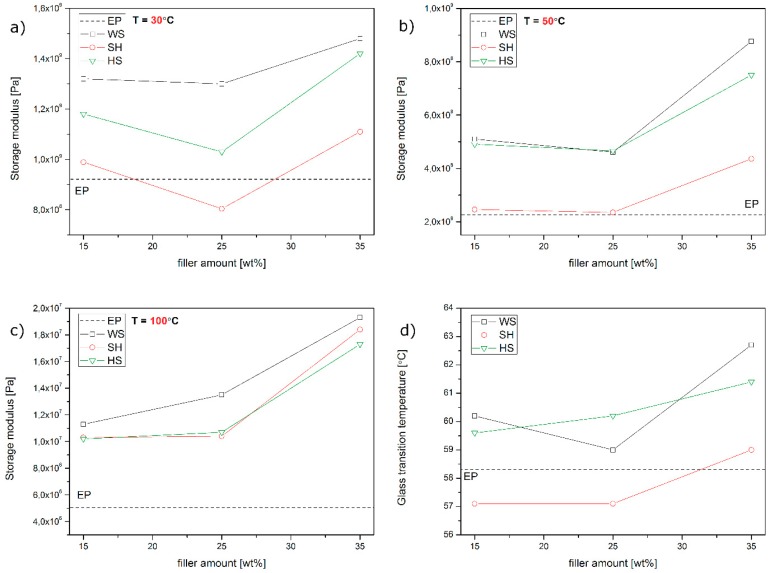
The values of composites storage modulus at various temperatures (**a**–**c**) and glass transition temperature (**d**).

In Figure 5, the results of the thermo-mechanical evaluation of the composites are presented. The values of the storage modulus (G’) and damping factor (tanδ) are presented on separate graphs as a function of temperature (T) measured for unmodified epoxy resin cast samples and epoxy-based composites that were filled with various amounts of the three analysed natural fillers. As can be seen in Figure 5a–c, the incorporation of all the natural lignocellulosic fillers that were used in this study caused an increase in the composites stiffness in almost the whole considered temperature range in comparison to the unmodified polymer. This effect may be attributed to the presence of rigid filler structures that are dispersed in a polymeric matrix, which allows the composite sample to accomplish an effective stress transfer despite the softening of the epoxy resin. This effect is due to the increasing molecular chains mobility at elevated temperatures. The only deviation from this tendency was observed for samples containing 25 wt % of the ground SH. In this case, the effect of stiffness improvement was observed above 48 °C, that is, at the beginning of the composite glass transition. Usually, the storage modulus vs. temperature curve of fully cured epoxy resin and its composites consists of three temperature-dependent distinct regions: a glassy region at a low temperature, an intensive drop of storage modulus corresponding to α-relaxation, and a rubbery plateau at a high temperature [23,24,25]. The extended stiffness of composite samples at a glassy region is an effect of effective reinforcement of the polymer by natural filler, which improves the epoxy resin’s ability to support the mechanical strains with recoverable deformation [24]. The comparison of the storage modulus values that were measured at selected temperature (30, 50, and 100 °C) is presented in Figure 6a–c. The highest storage modulus was observed for the composites that were modified with ground WS. As was described in our previous work, the incorporation of this filler type into the epoxy resin provides a significant modification of the mechanical properties of the composites [26]. An increase in G’ value is a simultaneous effect of the filler stiffness, which enables an effective stress transfer during the load and interfacial bonding between the composite components [16]. The lowest stiffness of the composites was observed for epoxy castings filled with SH, and the biggest difference was observed at 50 °C. Those results are reasonable if they are discussed in reference to the measured composites glass transition temperature (Figure 6d). The glass transition temperature (T_g_) of epoxy-based composites was determined at a peak of tanδ vs. T curves presented in Figure 5d–f. Detailed information regarding Tg is presented in the aforementioned Figure 6d. The incorporation of ground WS and HS results in an increase in the epoxy-based composites glass transition temperature, whereas the opposite tendency was denoted in the case of SH-filled composites. This phenomenon may be attributed to the chemical structure of the filler, as well as the shape of ground organic particles. The main difference in the chemical structure of the fillers used in this study is connected to the amount of lignin and fat [21]. Lignin is responsible for lingo-cellulosic material stiffness. A higher amount of lignin in organic fillers (HS 38 wt % and WS 35 wt %) results in an increased stiffness of those fillers in comparison to SH, which only contains 22 wt % of lignin [20]. What is interesting, the self-standing lignin that is incorporated into the polymeric matrix as a filler may be effective plasticizing agent [27,28]. Figure 6d shows the different effects of various organic fillers added to epoxy-based composites, whose glass transition temperature is mostly connected with the amount of fat in the agricultural waste plant fillers: the higher amount of fat, the lower glass transition temperature. In the case of SH composites that contain 8% of fat [21], glass transition temperatures were the lowest. The tendency in glass transition changes that were evaluated by DMA are in a good agreement with the experiments conducted with differential scanning calorimetry (DSC) described in our previous work [17]. It cannot be excluded that the observed lowered T_g_ in the case of SH-filled composites is partially connected with insufficient adhesion between the polymeric matrix and the filler, as well as increased porosity that is caused by hindered degassing in case of SH composites containing short fibre-shaped fillers. However, due to the observation of glass transition reduction effect by two different methods it can be stated that the dominant effect in the case the thermo-mechanical properties modification of SH-filled composites is caused by residual fat and oil migration. Effective plasticization of the polymer usually results in a simultaneously observed decrease of the T_g_ and an increase in the tanδ value at the peak as a result of the increased damping properties of the modified material. High amounts of fat in SH, which partially migrate from the organic particle to an interfacial region, provide a limitation in reactivity between the organic filler and the thermoset polymeric matrix, which may occur during the curing [26]. Higher values of the tanδ that were measured at α-relaxation may be related to poorer interfacial bonding between the matrix and the filler, which results in a higher mechanical energy dissipation [23]. On the basis of our previous studies concerning the effect of the filler shape, as well as migration of the fat and oil from agricultural filler on various polymers [17,29,30], it can be concluded that the reinforcing effect in the thermo-mechanical behaviour of the composites containing particle-shaped fillers may be suppressed by oil migration, resulting in simultaneously occurring plasticization of the polymeric matrix as well as the accumulation of oil in the interfacial region. In the case of SH-filled composites for all compositions, a significant reduction in the damping behaviour was noted, which, in reference to the previously discussed observations regarding the composite structures and the shape of the fillers, may be attributed to a different behaviour of the filler during the high shear mixing and the confined gas micro domains removal before forming.

### 3.4. Thermal Stability Analysis

**Figure 7:**
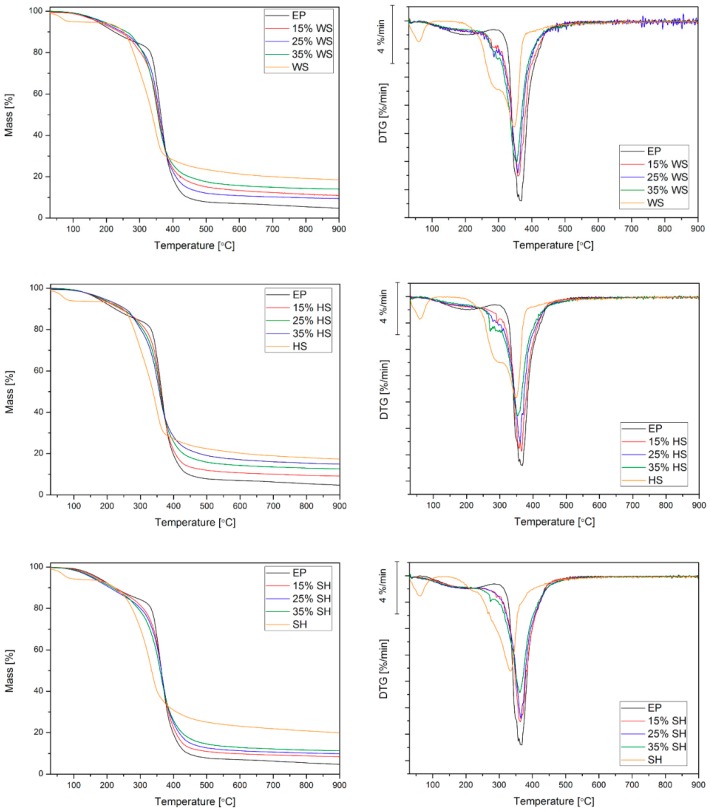
TG and DTG curves of epoxy resin, epoxy composites and natural filler investigated in an inert atmosphere.

The impact of the applied plant fillers on the thermal stability of polymer composites was evaluated through TGA. The values that were read from the curves of mass loss (TG) and derivatives of mass loss (DTG) are presented in Table 1 and Table 2, while the graphs are shown in Figure 7. The peaks appearing on DTG curves illustrate the rate of changes occurring in the materials and provide information regarding their chemical structure. As a result of the temperature increase, the dehydration process occurs first, accompanied by the emission of liquid and volatile compounds [12,13,14,15]. Based on the mass change in the range from ambient temperature up to 130 °C, it was observed that the amount of water in the fillers could be as much as about 3% (Table 1). It is important to determine the degradation temperature of the plant waste fillers (T_dmax_) due to the increase in temperature in the course of the composites production related to mechanical mixing and the cross-slinked epoxy resin process. The T_dmax_ is the value that corresponds to a mass loss equal to 1% at a temperature above 150 °C, below which the entire water present in the component evaporates [30]. The obtained values were about 220, 215, and 195 °C in the case of HS, WS, and SH, respectively, and they were much higher when compared to the temperature, which may be recorded during the manufacturing of the composites. The peaks that were observed for HS and WS at a temperature of about 290 °C, being difficult to determine in the case of SH, correspond to the decomposition of hemicellulose and the glycosidic linkage of cellulose. The next ones occurring at a temperature of about 350 °C correspond to the degradation of α-cellulose [31]. The lignin decomposition gradually occurs in a wide range of temperatures and quite often overlaps with the degradation of the other compounds without a sharp peak [32,33,34,35]. The residual that was determined at 900 °C reached approximately 20% for all of the tested fillers, and the largest yield was obtained for ground SH. Similarly, tests that were carried out in the air were conducted to ascertain the amount of residue and allow for calculating the share of organic components. In the case of the tested fillers, the amount of organic constituents ranged from 91% to 94%. Peaks that are present on the derivative curves of the mass loss of the composites correspond to the degradation processes of the organic fillers’ components, i.e., hemicellulose (270–300 °C) and α-cellulose (330–370 °C) [27,28,29,30,31]. However, the peak that was associated with the EP degradation (which occurred at 368 °C) was difficult to distinguish on the DTG curves, as it coincided with cellulose decomposition. It was observed that the transformation was shifted towards lower temperatures as the amount of plant filler (and hemicellulose) in the composites increased (Table 2).

The thermal stability of the composites was defined on the basis of temperatures corresponding to 5%, 10%, and 50% mass loss read from the TG curves. The onset temperature of degradation (5% mass loss) was subject to growth that was correlated with the increase in the amount of plant component, and the highest value (higher by 18 °C when compared to EP) was determined for the composites containing 35 wt % WS and 35 wt % HS. Different results were only noted in the SH series, for which the temperature was lower when compared to EP and decreased as the content of husk increased, which is probably related to the largest contribution of secondary components, i.e., fats and protein [21]. The temperature of 5% mass loss was above 150 °C, regardless of the type and amount of the natural component, which is higher than the expected temperature of utilization of the final products. Based on the analysis of the values corresponding to 10% mass loss (Table 2) and the course of TG curves of composites with shell (Figure 7), it was found that the materials have higher thermal stability in the temperature range up to 300 °C when compared to the unmodified epoxy resin. Probably, the improved thermal stability of the composites was related to the modified curing process of epoxy composites, as described in our previous work [16]. However, in the case of the temperature corresponding to 50% mass loss, the values that were obtained for the resin and composites were similar, which is probably related to the intense degradation of all the components (including the polymer matrix) at this stage of the test. Interestingly, slightly higher values were obtained for the SH series among the composites, unlike in the case of temperatures corresponding to 5% and 10% mass loss.

The differences in the decomposition rates were associated with the amounts of shell and husk in composite materials. Although the peak values determined for composites were lower when compared to EP (Table 2), the intensity of the transformation was reduced as a function of increasing filler content. Similarly, the residue at 900 °C grew with increasing organic compounds and it corresponded to the amount of lignin that they contained (SH < WS < HS) [21]. This is associated with the presence of aromatic groups in lignin, the occurrence of which promotes the formation of char [12,13,14,15].

### 3.5. Flammability Behaviour

**Figure 8:**
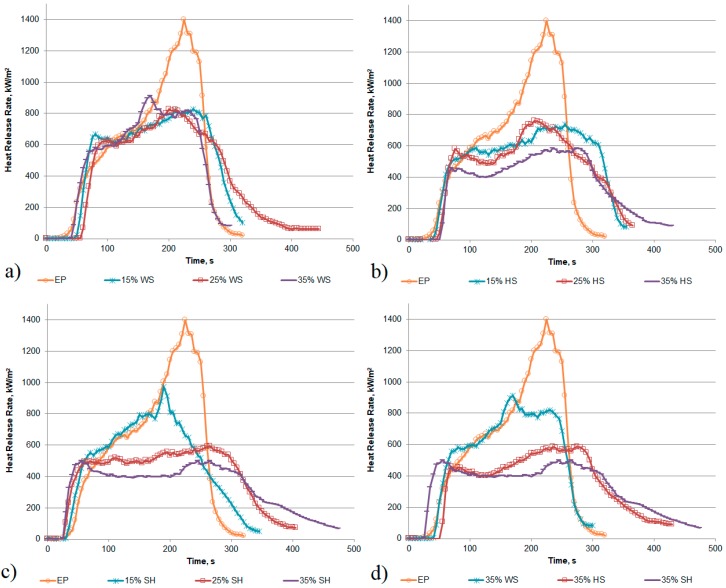
Effects of plant waste filler on the heat release behaviour of epoxy resin composites: (**a**) 15–35% WS; (**b**) 15–35% HS; (**c**) 15–35% SH; (**d**) 35% WS, HS and SH.

Heat Release Rate (HRR) is the single most important variable to evaluate fire safety of materials [36]. The representative heat release curves of epoxy resin and epoxy-based composites that were filled with different amount of shell or husk are presented in Figure 8. It was found that the curves of unmodified polymer were characterized by one single peak with the value of 1156 kW/m^2^, while the HRR of composites in most cases showed a broad shoulder without a well-defined peak. The incorporation of plant waste fillers into the epoxy matrix caused a decrease in the peak of Heat Release Rate (pHRR) values in comparison to the polymer (Table 3). For instance, the values of pHRR for sunflower pellets reached 192 kW/m^2^ in the article [37], which is much lower when compared to epoxy resin. The application of 15 wt % of less combustibility filler caused a slight reduction in the heat release rate, while increasing its amount by additional several percent resulted in a sharp reduction of the analysed parameter. For composite materials that were modified with 35 wt % of the ground sunflower husk the pHRR value, mainly about 520 kW/m^2^, was almost twice lower in comparison to the EP sample (Figure 8d). The addition of the filler causes a reduction in the pHRR values, which is attributed to the formation of carbonaceous char (approx. 14% fire residue from the initial mass in the case of composites with the highest amount of the filler). The lack of a clear dependence, according to which the pHRR values linearly decrease with the increase in SH share, results from the fact that it is not a commercial product with strictly defined composition and properties. Moreover, only a larger amount of filler causes the formation of char, which covers a significant part of the sample and it can effectively protect the polymer.

Simultaneously, the values of the Total Heat Release (THR), which is also an important parameter for evaluating flame retardancy of a material [38,39], were lower for composites in comparison to the EP. The Maximum Average Rate of Heat Emission (MAHRE) makes it possible to foresee fire development in full scale conditions. It can be clearly observed that the shell or husk incorporation led to a reduction in MAHRE for all the composites and for most series the values decreased as a function of increasing filler content. 

The parameters that evaluate the smoke behaviour according to the conducted studies using the cone calorimeter are Specific Extinction Area (SEA) and Total Smoke Release (TSR). When comparing the values of SEA obtained for the epoxy resin with the results that were determined for composites, it was found that as little as 15 wt % of the filler resulted in a fumes emission decrease by 46%. The lowest result was denoted for the composite containing 35 wt % of HS (642 m^2^/kg), while the value was 1582 m^2^/kg in the case of the unmodified polymer. The lowest value of the Total Smoke Release was also achieved for 35 wt % HS and it was more than two and a half times lower in comparison to the unmodified resin.

**Figure 9:**
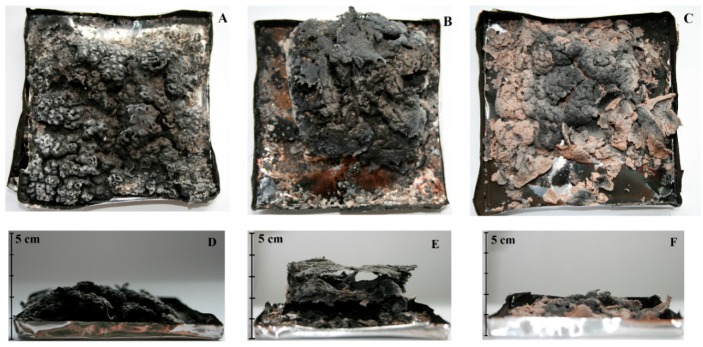
Photographs of selected materials after a cone calorimetry tests: 35 wt % WS (**A**,**D**), 35 wt % HS (**B**,**E**), and 35 wt % SH (**C**,**F**).

The photographs of the cone carbonaceous char that are presented in Figure 9 illustrate the burning behaviour differences between composites with the highest content of plant waste fillers. In contrast to the completely burned epoxy resin, the cone residue formed in the case of all composites allowed for ascertaining that the introduction of a natural filler led to the formation of a char layer. It was observed that the amount, as well as the type of the filler, determined the yield and appearance of char. The photograph of composites containing ground hazelnut shell allowed for observing the residues in the form of swollen char shaped in multicellular layers (Figure 9B,E). The 35 wt % WS composite gives a dense carbonaceous char after complete burning, but only with a small volume gain (Figure 9A,D). Meanwhile, an integrated char with holes covering the whole container was noticed in the case of the epoxy resin that was modified with SH (Figure 9C,F). Probably, the chemical structure of organic compounds, in particular the presence of lignin and cellulose that promote the formation of carbonaceous char, had influence on the quality of the residue. Moreover, the hemicellulose consisted of various saccharides (xylose, glucose, galactose, etc.) and it degraded to volatiles evolving out (including CO and CO_2_) [40], which favoured the formation of a cellular structure of char. We cannot exclude the possibility that the fine-grained form of particles in the case of shell boosts the raising of the solidifying layer of char.

**Figure 10:**
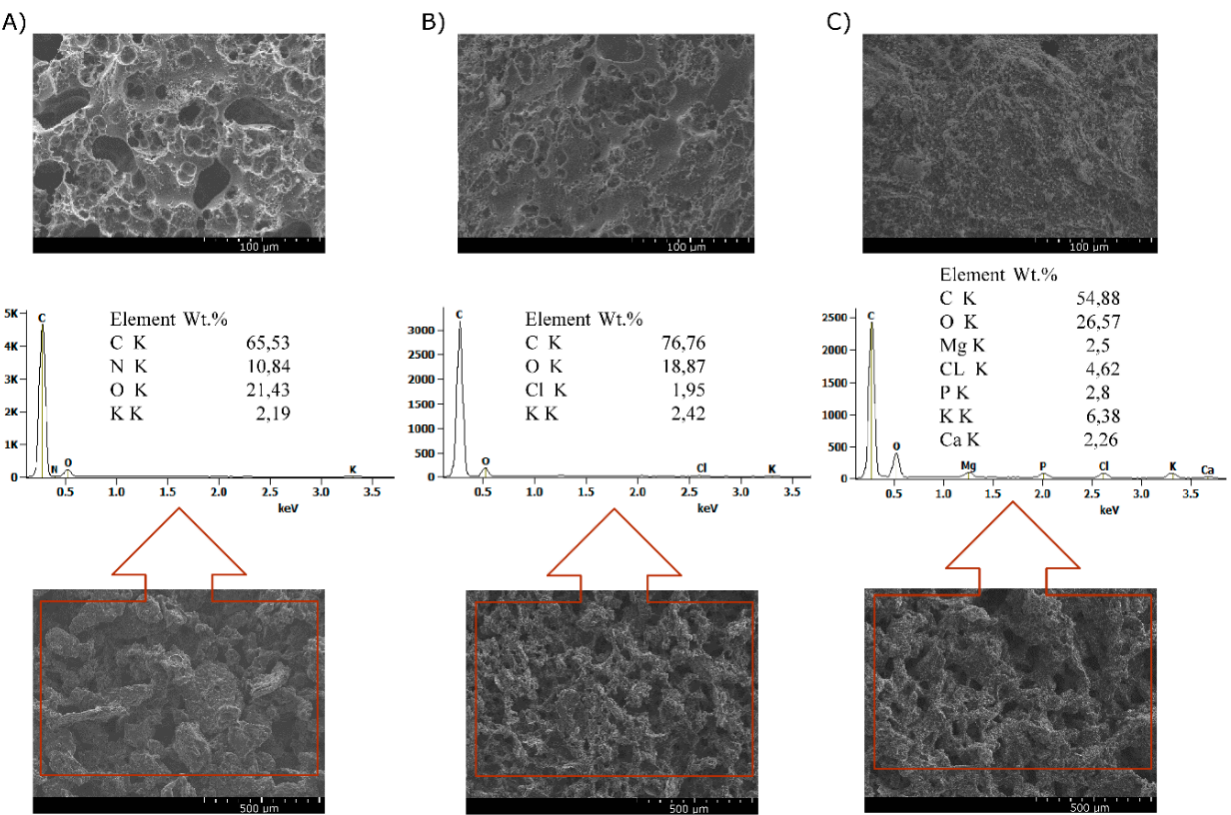
SEM images of the char residue of 35 wt % WS (**A**), 35 wt % HS (**B**), 35 wt % SH (**C**) (top—magnification ×500 and bottom—magnification ×100).

The morphology of the samples after cone calorimetry tests was investigated by SEM images analysis. The porous and swollen structure of all char, being especially visible in the inner part, confirms that ground shell and husk act similarly to intumescent flame retardants. Figure 10a,b allow for observing quite smooth surface of the char with a lot of holes, while that of 35 wt % SH is rougher and continuous (Figure 10c). The formation of swollen char with a solid top layer, covering the whole surface of the materials, ensures the effectiveness of char barrier. The chemical composition showed that the chars comprised C, O, and K, but also N, P, and Cl elements. This implies that the chemical structures of shell and husk decomposed into oxides and other products of degradation, which participated in the formation of a cross-linking network creating char [41]. It cannot be excluded that some of the elements were nothing but contamination.

### 3.6. Purser Furnace—GC-MS Analysis

**Figure 11:**
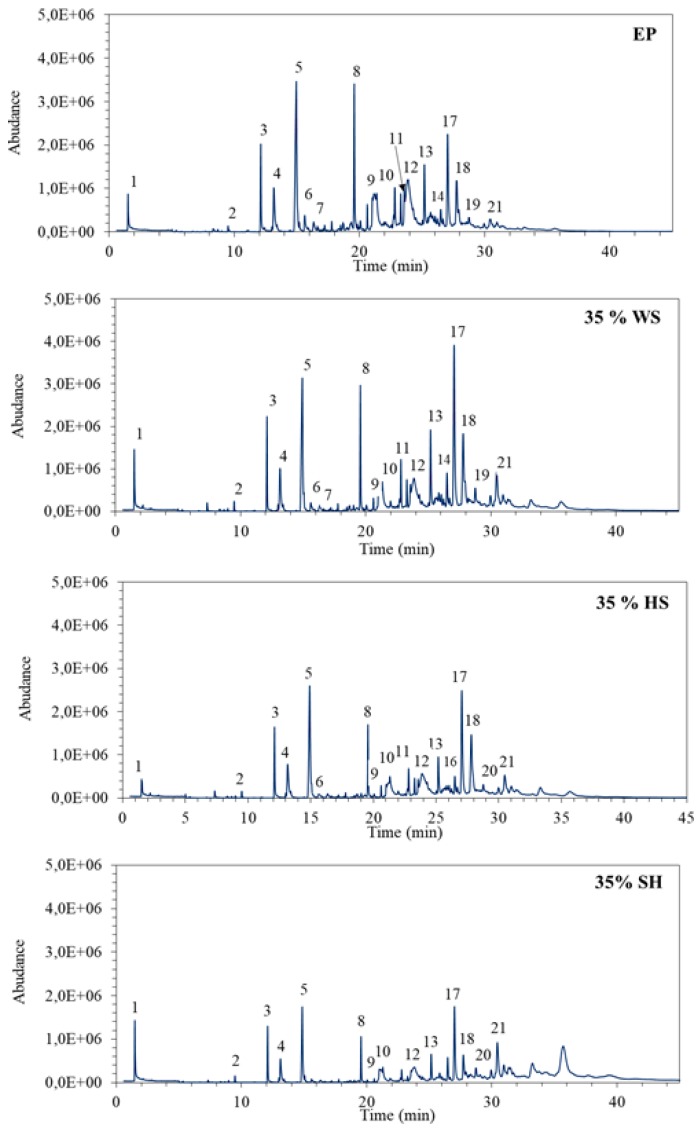
Chromatograms from analysis of thermal degradation products of selected modified epoxy composites at 650 °C.

The results that were obtained while using a Purser furnace and a GC-MS show that the amount and number of products released during the degradation of selected composites depend on the type of agricultural waste material used for the modification (Figure 11). The materials designated as 35 wt % WS behaved similarly to the unmodified epoxy resin (EP), while a significant decrease in the quantity and number of emitted products was observed for composites with HS and SH (29 and 26 compounds were detected in the samples of thermal degradation products of composite with 35 wt % HS and 35 wt % SH). However, the significant increase in the non-flammable production (COx, NOx, and H2O), diluting the oxidant in fire zone and inhibiting the combustion process, was recorded for composite with 35 wt % WS.

Table 4 shows the main compounds that were identified in the samples of gases and fumes emitted during the thermal degradation of selected polymers. In the largest quantities, polycyclic aromatic hydrocarbons, and phenolic compounds (such as: phenol, 2-methylphenol, 3-methylphenol and isopropenylphenol) were formed, especially when the test materials were EP and 35 wt % WS. At the same time, the addition of HS and SH reduced the amounts and types of evolved products, especially phenols, which are highly toxic pollutants. The smallest number of substances was detected and identified during the thermal decomposition of composites with 35 wt % SH.

## 4. Discussion

Composite materials that were modified with walnut and hazelnut shell, as well as sunflower husk, were prepared and examined. The incorporation of all the natural lignocellulosic fillers that were used in this study caused an increase in composite stiffness in comparison to the unmodified polymer, which may be attributed to the presence of rigid filler structures dispersed in the polymeric matrix. Based on the thermogravimetric analysis, it was found that the composites have a higher thermal stability in the temperature range up to 300 °C when compared to the epoxy resin. The intensity of the degradation reduced as a function of increasing filler content, while the yield of residue corresponded to the amount of lignin that was contained in the plants. 

The incorporation of plant waste filler into the epoxy resin matrix led to a modification in the fire and smoke behaviour of the polymer matrix. Materials containing fillers in the form of ground shell and husk showed a decrease in heat and fumes release in comparison to the unmodified EP. The formation of less combustible char layer inhibited the decomposition process of composites and limited the smoke emission. It was observed that the yield of char was determined by the amount and type of the filler. Composites containing ground hazelnut shell showed the residue in the form of swollen char shaped in multicellular layers.

Using a steady state tube furnace and a gas chromatograph with mass spectrometer allowed for identifying the major organic substances present in the gases and fumes that were emitted during the combustion of selected epoxy composites with agricultural waste. It was found that the addition of agricultural waste (especially hazelnut shell as well as sunflower husk) reduces the amount and the number of emitted products, and also determines their type.

## Figures and Tables

**Table 1:** Values determined based on thermogravimetric (TG) analysis conducted for WS, HS, and SH in nitrogen as well as air (organic components).

Filler	Water, %	T_150+1%_, °C	DTG 1, °C	DTG 2, °C	Residual Mass at 900 °C, %	Organic Components, %
WS	3.1	216	291	345	17.9	94.1
HS	3.8	215	290	347	17.8	94.0
SH	3.8	194	-	333	19.7	91.1

**Table 2:** TG and DTG data of epoxy resin (EP) and composites investigated in an inert atmosphere.

Sample Designation	5% Mass Loss, °C	10% Mass Loss, °C	50% Mass Loss, °C	DTGA, °C, %/min	Residual Mass 900 °C, %
EP	172	224	364	368, −12.9	4.74
15% WS	183	247	360	358, −11.0	9.43
25% WS	175	240	357	358, −10.8	10.96
35% WS	190	261	355	354, −10.0	13.98
15% HS	178	240	361	365, −11.8	9.15
25% HS	181	245	360	361, −11.1	12.66
35% HS	190	255	357	354, −9.1	14.96
15% SH	167	223	363	363, −11.1	8.50
25% SH	154	209	364	365, −10.8	9.90
35% SH	158	215	362	362, −8.9	11.31

**Table 3:** Cone calorimeter results of epoxy composites modified with shell and husk (standard deviation).

Sample Designation	TTI,	pHRR,	THR,	MARHE,	Fire Residue,	SEA,	TSR,
	s	kW/m^2^	MJ/m^2^	kW/m^2^	%	m^2^/kg	m^2^/m^2^
EP	51(17)	1156 (221)	184 (7)	569 (82)	8 (1)	1582 (554)	10,353 (3672)
15% WS	47 (6)	803 (81)	167 (1)	536 (43)	10 (1)	834 (43)	5303 (284)
25% WS	55 (2)	826 (14)	167 (7)	531 (12)	11 (2)	743 (22)	4731 (239)
35% WS	46 (3)	873 (87)	155 (2)	543 (26)	14 (0)	650 (35)	3986 (199)
15% HS	46 (1.5)	744 (92)	158 (18)	502 (39)	11 (1)	849 (22)	5122 (490)
25% HS	47 (3)	802 (78)	161 (1)	487 (33)	12 (0)	810 (58)	4974 (365)
35% HS	50 (3)	677 (81)	143 (2)	428 (27)	14 (1)	642 (17)	3915 (147)
15% SH	37 (2)	1002 (150)	161 (4)	568 (43)	11 (0)	857 (9)	5344 (184)
25% SH	38 (11)	615 (62)	158 (2)	447 (28)	11 (1)	700 (114)	4368 (713)
35% SH	31 (5)	520 (69)	151 (2)	383 (21)	14 (1)	716 (13)	4371 (127)

**Table 4:** Thermal degradation products of selected modified epoxy composites at 650 °C.

Peak Number	Retention Time (min)	Compound	Emissionyields (peakarea %)			
			EP	35 wt % WS	35 wt % HS	35 wt % SH
1	1.50	CO_x_, NO_x_, H_2_O	1.60	2.51	1.51	4.41
2	9.49	Styrene	0.16	0.33	0.39	0.39
3	12.11	Benzaldehyde	3.93	3.74	4.67	3.82
4	13.15	Phenol	3.67	3.96	4.47	3.12
5	14.95	Benzylalcohol	16.04	12.29	17.31	8.99
6	15.62	2-methylphenol	1.14	0.60	0.67	
7	16.33	3-methylphenol	0.93	0.68		
8	19.58	Naphthalene	7.11	5.34	4.97	3.19
9	21.16	2,3-dihydro-benzofuran	5.95	3.01	2.49	2.73
10	22.82	1-methylnaphthalene	2.46	2.11	1.99	0.86
11	23.29	2-methylnaphthalene	1.67	1.24	1.28	
12	23.87	p-isopropenylphenol	13.82	8.20	9.12	5.96
13	25.18	Biphenyl	4.07	4.66	3.57	2.48
14	25.83	1,3-dimethyl-naphthalene	1.07	1.08		
15	26.03	Ethenylnaphthalene	0.68	0.77		
16	26.68	1,2,3,4-tetrahydro-2,5,8-trimethyl-1-naphthalenol	0.49	0.57	0.73	
17	27.04	Acenaphthylene	8.13	14.12	14.17	10.20
18	27.56	1-dodecanol	4.63	6.92	11.15	3.53
19	27.92	1-isopropyl-naphthalene	1.44	1.78		1.13
20	28.74	Dibenzofuran	0.67	1.22	0.82	1.24
21	30.44	Fluorene	1.78	4.70	4.51	7.77
